# The opinion of clinical staff regarding painfulness of procedures in pediatric hematology-oncology: an Italian survey

**DOI:** 10.1186/1824-7288-37-27

**Published:** 2011-06-10

**Authors:** Chiara Po', Franca Benini, Laura Sainati, Anna C Frigo, Simone Cesaro, Maria I Farina, Caterina Agosto

**Affiliations:** 1 Pediatric Pain and Palliative Care Service, Department of Pediatrics, University of Padua, Italy; 2 Clinic of Pediatric Hematology-Oncology, Department of Pediatrics, University of Padua, Italy; 3 Department of Environmental Medicine and Public Health, University of Padua, Italy; 4 Pediatric Hematology-Oncology Unit,"G.B. Rossi" Hospital, Verona, Italy

## Abstract

**Background:**

Beliefs of caregivers about patient's pain have been shown to influence assessment and treatment of children's pain, now considered an essential part of cancer treatment. Painful procedures in hematology-oncology are frequently referred by children as the most painful experiences during illness. Aim of this study was to evaluate professionals' beliefs about painfulness of invasive procedures repeatedly performed in Pediatric Hemato-Oncology Units.

**Methods:**

Physicians, nurses, psychologists and directors working in Hemato-Oncology Units of the Italian Association of Pediatric Hematology-Oncology (AIEOP) were involved in a wide-nation survey. The survey was based on an anonymous questionnaire investigating beliefs of operators about painfulness of invasive procedures (lumbar puncture, bone marrow aspirate and bone marrow biopsy) and level of pain management.

**Results:**

Twenty-four directors, 120 physicians, 248 nurses and 22 psychologists responded to the questionnaire. The score assigned to the procedural pain on a 0-10 scale was higher than 5 in 77% of the operators for lumbar puncture, 97.5% for bone marrow aspiration, and 99.5% for bone marrow biopsy. The scores assigned by nurses differed statistically from those of the physicians and directors for the pain caused by lumbar puncture and bone marrow aspiration. Measures adopted for procedural pain control were generally considered good.

**Conclusions:**

Invasive diagnostic-therapeutic procedures performed in Italian Pediatric Hemato-Oncology Units are considered painful by all the caregivers involved. Pain management is generally considered good. Aprioristically opinions about pain depend on invasiveness of the procedure and on the professional role.

## Background

Awareness and knowledge about children's pain increased substantially in the last three decades. Many studies have demonstrated that treatment-related pain and procedural pain are often the worst causes of pain in children with cancer[1,2]. Young patients in fact often refer to lumbar puncture and bone marrow aspiration (procedures repeatedly conducted as part of diagnostic and therapeutic protocols) as the most painful experiences relating to their malignancy[2]. This experience is associated not only with the invasiveness of the procedures, but also with the children's fear of needles[3] and memories of previous procedures, which influence their reaction to subsequent procedures[4-6]. The child's life-long quality of life may be affected by these painful experiences[7].

Therefore procedural pain treatment is now considered an essential part of cancer patient's care, and recommendations were implemented in many nations[8-14].

Beliefs and attitudes of caregivers about patient's pain have been shown to influence assessment and treatment of children's pain[15].

Aim of this study was to estimate the beliefs of healthcare professionals about painfulness of the repeated invasive diagnostic-therapeutic procedures in Pediatric Hemato-Oncology Centers of the AIEOP network (Italian Association of Pediatric Hematology Oncology). A further objective was to evaluate whether or not personal and organizational factors could influence clinical staff's belief about pain. We also evaluated healthcare professionals' opinions about pain management in their own Center and about relevant factors that discourage use of sedation analgesia.

## Methods

### Participants

We developed a nationwide survey among the Italian Association of Pediatric Hematology-Oncology (AIEOP), which includes the centers treating more than 90% of pediatric cancer patients in Italy. All the healthcare professionals working in each Centers were involved, including physicians, nurses, psychologists and directors.

### Questionnaires

The survey focused on the three invasive procedures (lumbar puncture, bone marrow aspiration and bone marrow biopsy) most frequently used in diagnostic and therapeutic protocols. The questionnaire (to be filled in anonymous format) [see additional file 1: The questionnaire] consisted of 4 closed questions:

1. to indicate professional role (physician, nurse, psychologist, director) and working age (under 5 years, 5-10 years, 10 years)

2. to indicate believed painfulness of the three procedures (lumbar puncture, bone marrow aspiration and bone marrow biopsy) on a 0-10 scale (0 = no pain, 10 = the worst pain)

3. to indicate opinion about pain management on a 0-10 scale (0 = the worst control, 10 = the best control)

4. to indicate on a Likert scale format from 0 to 4 (0 no importance, 4 maximum importance) the relevance of: shortage of time, lack of space and equipment, lack of adequate training, shortage of dedicated staff for managing sedation-analgesia, doubts about the safety of sedation-analgesia to determine the decision to perform procedures without sedation-analgesia.

The number of procedures carried out annually at each center (which we used as a dimensional parameter) and the number of physicians, nurses and psychologists on the staff were asked to the Director of each center.

Questionnaires and instructions were sent to Directors of each center in April, 2010. The deadline for returning completed questionnaires by all the healthcare professionals was by September, 30 2010.

### Statistics

The results are reported as counts and percentages for the categorical variables (scores globally assigned to the pain and to its control), as quartiles, minimum and maximum for the ordinal ones (distribution of ratings assigned to procedural pain).

The number of lumbar punctures, bone marrow aspirates and biopsies was dichotomized according to median value (respectively 150-150-70); total number of procedures was dichotomized according to clinical consensus (500). The comparison between the number of procedures dichotomized was drawn using the Wilcoxon rank sum test.

The pain scores attributed by the different types of professional and the number of years spent in their professions were compared with the Kruskal-Wallis test followed by the pairwise comparison with the Dunn procedure[16] when the test result was statistically significant at the 5% level. The analyses were conducted with the SAS rel. 9.1 for Windows (SAS Institute Inc., Cary, NC, USA).

## Results

Twenty-four of the 54 centers contacted responded to questionnaire (44.4%).

In these centers the number of procedures handled per year ranged from 50 to 1,565 and 33% of the centers performed more than 500 procedures/year. The responders were the directors of the 24 Centers, 120 physicians, 248 nurses, 22 psychologists, representing respectively: 48% of physicians, 52% of nurses, 42% of psychologists employed in the 24 Centers which responded.

### Beliefs about pain

**Figure 1:**
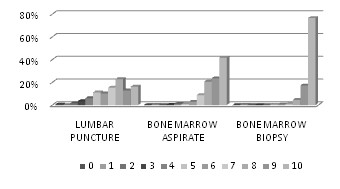
**Believed painfulness of procedures**. Distribution of scores assigned by all the healthcare professionals on a 0-10 scale to believed painfulness of lumbar puncture (a), bone marrow aspiration (b), and bone marrow biopsy (c)

The score assigned on a scale of 0-10 exceeded 5 in 77.2% for the lumbar puncture, 97.5% for the bone marrow aspirate, 99.5% for the bone marrow biopsy [Figure 1].

Physicians, nurses, psychologists and directors scored differently the pain caused by lumbar puncture (p = 0.0001) and bone marrow aspiration (p < 0.0001), while there were no significant differences for bone marrow biopsy (p = 0.1354).

**Figure 2:**
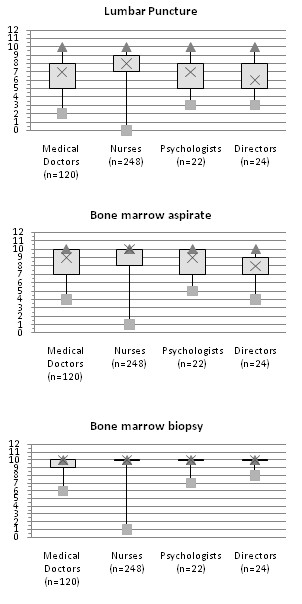
**Painfulness scored by different professional categories**. Distributions of scores assigned by different professional categories on a 0-10 scale to believed painfulness of lumbar puncture (a), bone marrow aspiration (b), and bone marrow biopsy (c) (min, max, quartiles; 0 = no pain, 10 = the worst pain).

In particular, we found a significant difference (at 5% level) concerning lumbar puncture and bone marrow aspirate comparing nurses with physicians and directors [Figure 2]. Nurses tended to attribute higher score to pain.

A correlation between operators' scoring of pain due to each procedure and the number of corresponding procedures performed annually in their own centers resulted significant for physicians about lumbar puncture and bone marrow aspiration (p = 0,0038 and p = 0,0002), for nurses about bone marrow aspiration (p = 0,0038), for psychologists about lumbar puncture and bone marrow aspiration (p = 0,0069 and p = 0,0212). The operators of centers performing a larger number of procedures tended to attribute lower pain scores whereas the responses of Directors seemed uninfluenced by the dimensions of the center.

Among nurses, a correlation between the pain score attributed to bone marrow biopsy and the number of years spent in the profession resulted statistically significant (p = 0.0005).

### Opinions about procedural pain management

**Figure 3:**
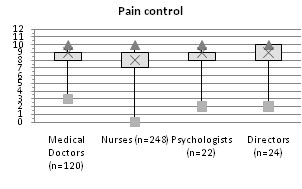
**Opinions about pain management**. Distributions of the opinions expressed by the different professional categories about pain management in their own center on a 0-10 scale (min, max, quartiles; 0 = the worst control, 10 = the best control).

The control of procedural pain performed at each center was generally rated highly. However physicians, nurses, psychologists and directors expressed heterogeneous opinions (p = 0.0037): pairwise comparisons demonstrated a statistically significant difference at the 5% level between physicians and nurses [Figure 3].

Operators' opinions were not affected by the number of procedures performed annually at each center (p = 0.3853 for nurses, p = 0.9087 for physicians, p = 0.9169 for psychologists, p = 0.4268 for directors) and by the years of professional experience (p = 0.2398 for nurses, p = 0.3864 for physicians, p = 0.5365 for psychologist).

### Opinion about factors discouraging use of sedation-analgesia

Among all responders, 60% of the nurses, 59% of the physicians, 77% of the psychologists, and 29% of Directors indicated the causes related to the performing the procedures without analgesia. The first relevant factors was the deficiency of dedicated staff for managing sedation-analgesia, relevant for 86% of nurses, 87% of physicians, 70% of psychologist, and 100% of Directors. This factor received the most highest score over all category: 42% of the nurses, 44% of the physicians, 29% of the psychologists, and 43% of the directors; the second most important reason was the doubts about the safety of sedation-analgesia (relevant for 65% of nurses), and following, the lack of space and equipment (relevant for 68% of the physicians and 72% of the directors), and the shortage of time (relevant for 65% of the psychologists).

## Discussion

A few studies investigated health professionals' concerns about pain in infants and children with cognitive impairment[17-20]. We would to evaluate hemato-oncology health professionals' beliefs about pediatric procedural pain.

We found that grade of painfulness attributed aprioristically to procedural pain is overall high in the opinion of all the professional categories investigated. Given that a pain scoring more than 4 on scale of 0-10 must necessarily be treated in clinical practice our findings reflect the importance attributed to procedural pain by all the operators involved in patient care. The Italian situation confirms the overall increasing attention to pain in children, which has spread among all operators in the last three decades[21,22]; a study comparing professionals' and children's opinions even demonstrated a certain tendency for professionals to overestimate pain in the pediatric oncology setting[23].

Painfulness attributed *a priori *to procedures seem to correlate with their invasiveness: barring a few cases, bone marrow aspiration and bone marrow biopsy were considered more painful than lumbar puncture. In particular, the scores attributed by all responders to the pain caused by bone marrow biopsy were homogeneously distributed around particularly high values (9 or 10/10).

Differences were found in the views expressed by the various professional categories: nurses' scores for pain due to lumbar puncture and bone marrow aspiration differed statistically from those of the physicians and directors, and were generally higher and more homogeneous. A such difference between nurses and physician was been already demonstrated[20,24]. A possible explanation is the professional different training, and relationships with patients: nurses establish a "caring" relationship with the children, looking after them throughout the course of their disease, spending a lot of time close to patients and their families, so they may have more opportunities to see the children's anxiety and fear, and the side effects of painful procedures. Physicians and directors focus on the patient's "treatment" with a view to achieving their "recovery", a picture in which invasive procedures represent a necessary step in the treatment protocol. Many studies confirmed Nurses' involvement in the "care": workload perceived too great to provide quality patient care appeared the most important cause of stress for nurses following one of their patient's death[25,26]; nurses administering chemotherapy felt the negative effects of a perceived decrease in their caring role, making them change from "nursing the patient" to "nursing the clinic"[27]. Nurses working routinely with children feel better when the children's pain is well controlled[28], and the amount of action they take on pediatric symptoms and their perceived effectiveness significantly influence nurses' levels of distress[29].

Unlike other experiences[20] pain management was generally considered good. Here again, however, nurses' opinions differed significantly from those of the physicians and psychologists, tending to be less optimistic.

Operators working in the centers performing yearly a great number of procedures attribute on average a lower believed painfulness to lumbar puncture and bone marrow aspirate, even if scores attributed to procedural pain persisted high. On the contrary, operators' opinion about pain management seems not to depend on the dimension of center. It means that an high workload is not perceived as a limit in providing an adequate pain management.

Sedation-analgesia is now globally considered the most important means for ensuring procedures without pain[9,12]. Opinions expressed by the various professional groups in our study on the reasons for not using sedation-analgesia were fairly homogeneous; in particular, the shortage of staff was generally considered highly relevant and more important than other issues, confirming the relevance of perceived workload in operators' opinions.

Physicians and directors attributed importance to the shortage of space more often than other professional categories, reflecting their attention to the organizational aspects of procedural pain management. Low relevance was attributed by all the professionals except the psychologists to the shortage of time, which prompts two considerations: the need to perform a large number of procedures does not seem to affect the way pain is managed, even though this takes time; psychologists see the shortage of time as a more important problem than the other operators considered because they need to establish a particular relationship with patients, which takes time, closeness and a quiet environment.

Nurses attributed greater relevance to doubts about the safety of sedation-analgesia, confirming their closeness to the children and consequently greater concern about the treatments they receive.

This study has an important bias in the fact it inquires an a priori opinion which could change in the actual situation, when each individual children undergoes the procedure. Nevertheless, we consider this survey an important step to depict state of the art of procedural pain management in Italy, because operators' beliefs about pain could influence pain assessment and treatment and contribute to establish a widespread awareness on pain in children.

A further limit of this study is that we received less answers than expected: less than 50% of contacted Directors sent questionnaires completed by their staff. Probably it could be difficult to collect completed questionnaires from working staff in the overwhelmed context of Pediatric Oncology Units. However the limited number of received responses suggests other substantial questions: had the management of procedural pain reached a so well-defined and widespread organization to be no longer considered an interesting issue? Or, on the contrary, discussion and education on procedural pain control need to continue to increase operators' awareness for the best practice? The importance of operators' continue education in the maintenance of quality standards is demonstrated[30]. So this study could be an opportunity to promote education on procedural pain control in pediatric oncologic field.

## Conclusions

In conclusion invasive diagnostic-therapeutic procedures performed in Italian Pediatric Hemato-Oncology Units are considered painful by all the healthcare professionals involved. Pain management is generally considered good.

Aprioristically beliefs about pain depend on invasiveness of the procedure and on the professional role; they are also partially influenced by the dimension of the centers. On the contrary they are not influenced by years of professional experience. Nurses attributed on average higher scores to pain and lower score to pain management, probably because of their main proximity to the child.

Staff shortages are perceived as the most important cause which prevents sedation-analgesia practice for painful procedures. Nurses attribute importance also to doubts about safety of pharmacological sedation.

So that, we want to highlight two possible optimization areas: first of them, a particular support should be assured to nurses who appear more influenced by workload and concerns about the patient in their role of "care". This support could include psychological support, stress management programs, educational programs about sedation-analgesia, and also a farsighted organizational policy (e.g. reducing working hours or time spent in contact with patients, increasing the nurse/patient ratio)[31]. Second, it should be important to educate all the caregivers about the extremely complex psycho-physical nature of pain experience, not necessarily related with the invasiveness of the procedure[32,33]; they should learn that all available means should be adopted to control every child's anxiety and pain, including both pharmacological and non-pharmacological treatments.

## Competing interests

The authors declare that they have no competing interests.

## Authors' contributions

CP wrote the paper. CA and FB supervised the study. CA, LS, FB coordinated the research. SC, LS and CA recruited the responders. CA and CP analyzed the data. MIF, CA, CP, SC and LS discussed the results. ACF provided statistical analysis and results elaboration. The authors confirm they have read and approved the final version of the manuscript.

## Supplementary Material

Additional files 1 **"Questionnaire investigating operators' beliefs about painful procedures"**. The file is the questionnaire (to be filled in anonymous format) investigating beliefs of operators about painfulness of invasive procedures (lumbar puncture, bone marrow aspirate and bone marrow biopsy) and level of pain management. Click here for file
