# Supplementary material for: The opinion of clinical staff regarding painfulness of procedures in pediatric hematology-oncology: an Italian survey
Source: Ital J Pediatr. 2011 Jun 10;37:27. doi: 10.1186/1824-7288-37-27 (PMC3127832; doi:10.1186/1824-7288-37-27)
Supplement: Additional files 1 — "Questionnaire investigating operators' beliefs about painful procedures". The file is the questionnaire (to be filled in anonymous format) investigating beliefs of operators about painfulness of invasive procedures (lumbar puncture, bone marrow aspirate and bone marrow biopsy) and level of pain management. [file 1824-7288-37-27-S1.DOC]

**Questionnaire**

Please,

provide the name of your Institution______________________________________________________

1) Please, indicate data about your professional role and working age:

Profession Working age

/_/ Physician /_/ less than 5 years

/_/ Nurse /_/ 5-10 years

/_/ Psychologist /_/ more than 10 years

/_/ Director

2) Please, indicate believed painfulness of these procedures (lumbar puncture, bone marrow aspiration, bone marrow biopsy) on a 0-10 scale (0 = no pain, 10 = the worst pain)

|  | 0 | 1 | 2 | 3 | 4 | 5 | 6 | 7 | 8 | 9 | 10 |
| --- | --- | --- | --- | --- | --- | --- | --- | --- | --- | --- | --- |
| LUMBAR PUNCTURE |  |  |  |  |  |  |  |  |  |  |  |
| BONE MARROW ASPIRATE |  |  |  |  |  |  |  |  |  |  |  |
| BONE MARROW BIOPSY |  |  |  |  |  |  |  |  |  |  |  |

3) Please, indicate your opinion about procedural pain management in your Center on a 0-10 scale (0 = the worst control, 10 = the best control)

| 0 | 1 | 2 | 3 | 4 | 5 | 6 | 7 | 8 | 9 | 10 |
| --- | --- | --- | --- | --- | --- | --- | --- | --- | --- | --- |

|  | **0** | **1** | **2** | **3** | **4** |
| --- | --- | --- | --- | --- | --- |
| Shortage of time |  |  |  |  |  |
| Lack of space and equipment |  |  |  |  |  |
| Lack of adequate training |  |  |  |  |  |
| Shortage of dedicated Staff for managing sedation-analgesia |  |  |  |  |  |
| Doubts about safety of sedation-analgesia |  |  |  |  |  |

4) Please indicate on the following Likert scale format from 0 to 4 (0 = no importance, 4 = max importance) the relevance of each items to determine the decision to perform procedures without sedation-analgesia
